# Correction to “Multifunctional Nanoregulator Reshapes Immune Microenvironment and Enhances Immune Memory for Tumor Immunotherapy”

**DOI:** 10.1002/advs.76073

**Published:** 2026-06-11

Meng Yu, Xiaohui Duan, Yujun Cai, Fang Zhang, Shuqi Jiang, Shisong Han, Jun Shen*, and Xintao Shuai*, “Multifunctional Nanoregulator Reshapes Immune Microenvironment and Enhances Immune Memory for Tumor Immunotherapy,” *Advanced Science* 6, no. 16 (2019): e1900037, https://doi.org/10.1002/advs.201900037.

The authors found that the pictures of the control group in Figure 4e (Row 2, Column 1) and Figure S10 (Row 2, Column 2) were incorrect. The corrected Figures with legends are provided. None of the conclusions of the main text are changed.

**FIGURE 4:**
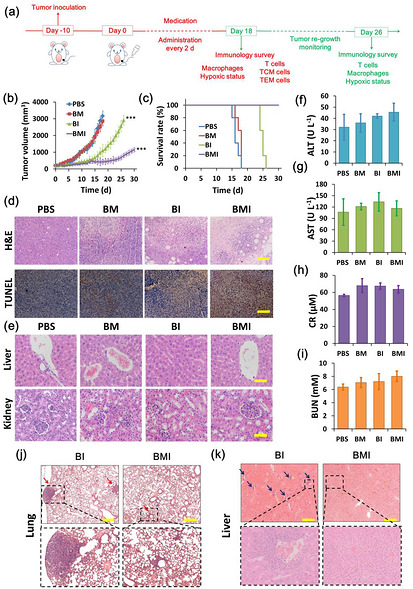
In vivo immunotherapeutic effect and toxicological studies of BMI administered via *i.v*. injection into 4T1 tumor‐bearing mice. (a) Schematic illustration of immunotherapeutic effect studies in vivo. Tumor‐bearing mice were tail vein injected with PBS, BM, BI, and BMI at a determined dose (2.5 µg of IPI549 or 10 µg of Mn per mouse) every 2 days during medication. (b) Tumor growth curves of mice after *i.v*. injection of different formulations (n = 5). Significance between the treatment groups and the control group during the medication treatment (0–18 d) was calculated using an unpaired two‐tailed Student's *t*‐test. ^*^ *p* < 0.05, ^**^ *p* < 0.01, ^***^ *p* < 0.001. (c) Survival rates of tumor‐bearing mice receiving various treatments (n = 5). (d) H&E and TUNEL analyses of 4T1 tumor tissue sections harvested at the time point 18 d from mice receiving various treatments. (e) H&E assay on liver and kidney tissues harvested at 18 d from various treatment groups. Scale bar: 100 µm. Serum levels of alanine transaminase (ALT), aspartate transaminase (AST), creatinine (CR), and blood urea nitrogen (BUN) in mice measured at 18 d during the course of treatment (f–i). H&E stained sections of the lungs (j) and livers (k) collected from 4T1 tumor‐bearing mice after drug withdrawal (i.e. at 26 d in Figure 4a).

**FIGURE S10:**
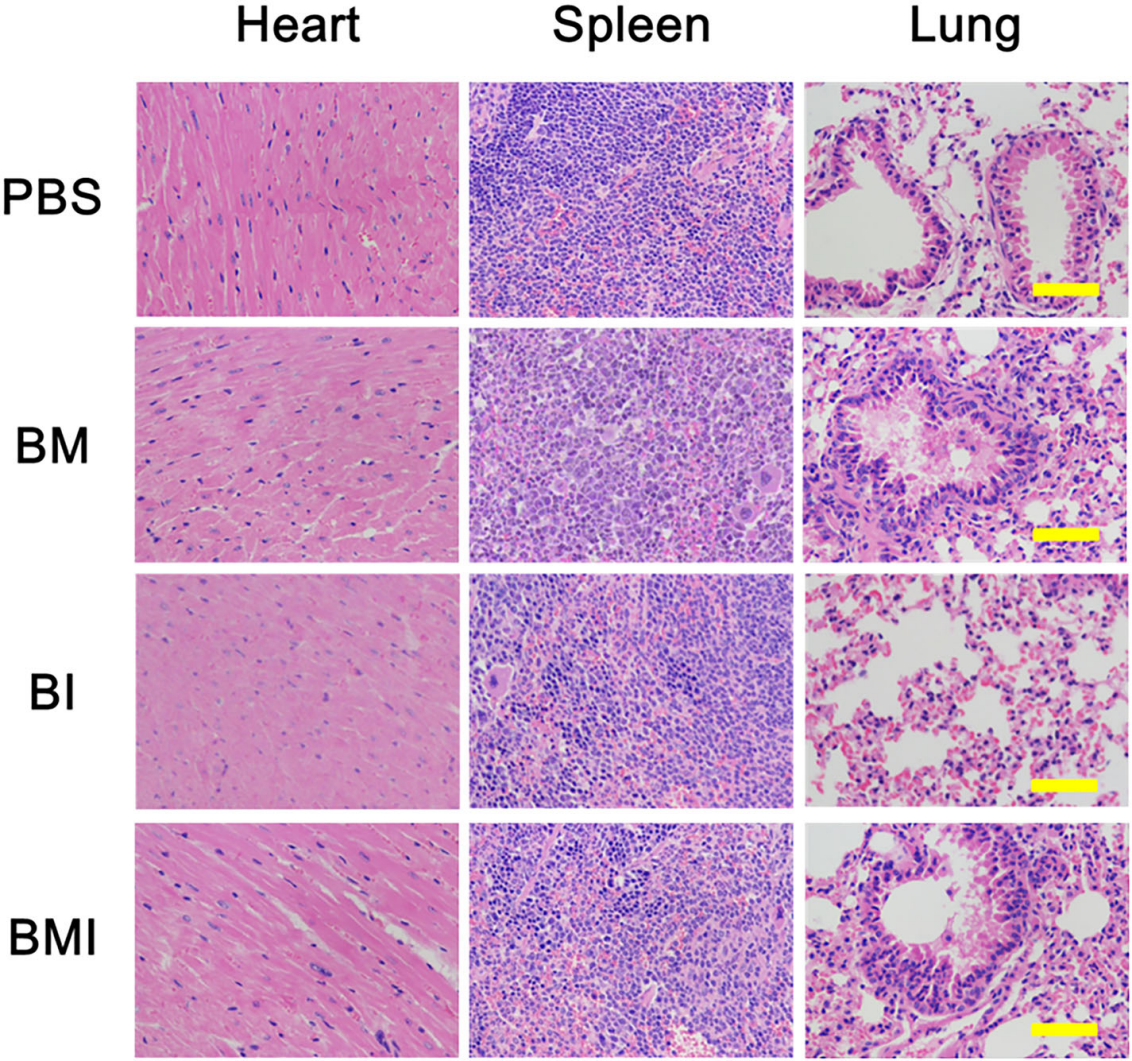
H&E staining for heart, spleen, and lung tissues from mice receiving various treatments at 18 d of treatment. Scale bar: 100 µm.

